# Trimethyl-Chitosan Coated Gold Nanoparticles Enhance Delivery, Cellular Uptake and Gene Silencing Effect of EGFR-siRNA in Breast Cancer Cells

**DOI:** 10.3389/fmolb.2022.871541

**Published:** 2022-04-20

**Authors:** Leila Baghani, Niloofar Noroozi Heris, Fatemeh Khonsari, Sajad Dinarvand, Meshkat Dinarvand, Fatemeh Atyabi

**Affiliations:** ^1^ Department of Pharmaceutical Nanotechnology, Faculty of Pharmacy, Tehran University of Medical Sciences, Tehran, Iran; ^2^ Department of Pharmaceutics, Faculty of Pharmacy, Tehran University of Medical Sciences, Tehran, Iran; ^3^ Nanotechnology Research Center, Faculty of Pharmacy, Tehran University of Medical Sciences, Tehran, Iran

**Keywords:** gold nanoparticles (AuNPs), trimethyl chitosan (TMC), EGFR-siRNA, siRNA delivery, breast cancer

## Abstract

**Purpose:** Despite the promising therapeutic effects of gene silencing with small interfering RNAs (siRNAs), the challenges associated with delivery of siRNAs to the tumor cells *in vivo*, has greatly limited its clinical application. To overcome these challenges, we employed gold nanoparticles modified with trimethyl chitosan (TMC) as an effective delivery carrier to improve the stability and cellular uptake of siRNAs against epidermal growth factor receptor (EGFR) that is implicated in breast cancer.

**Methods:** AuNPs were prepared by the simple aqueous reduction of chloroauric acid (HAuCl_4_) with ascorbic acid and coated with synthesized TMC. EGFR-siRNA was then complexed with the AuNPs-TMC *via* electrostatic interaction to make AuNPs-TMC/EGFR-siRNA with a w/w ratio of 10:1. Nanoparticles were assessed for physicochemical characteristics and *in vitro* cellular behavior on MCF-7 breast cancer cell line.

**Results:** Spherical and positively charged AuNPs-TMC (67 nm, +45 mV) were successfully complexed with EGFR-siRNA (82 nm, +11 mV) which were able to retard the gene migration completely. Confocal microscopy and flow cytometry analysis demonstrated complete cellular uptake of Cy5 labeled AuNPs-TMC in the MCF-7 cells after 4 h incubation. MTT test after 48 h incubation showed that the AuNPs-TMC were safe but when combined with EGFR-siRNA exert significant cytotoxicity while the cell viability was about 50%. These nanocomplexes also showed a high gene expression knockdown (86%) of EGFR and also a high apoptosis rate (Q2 + Q3 = 18.5%) after 24 h incubation.

**Conclusion:** This study suggests that the simply synthesized AuNPs-TMC are novel, effective, and promising nanocarriers for siRNA delivery, and AuNPs-TMC/EGFR-siRNA appears to be a potential therapeutic agent for breast cancer treatment.

## Graphical Abstract

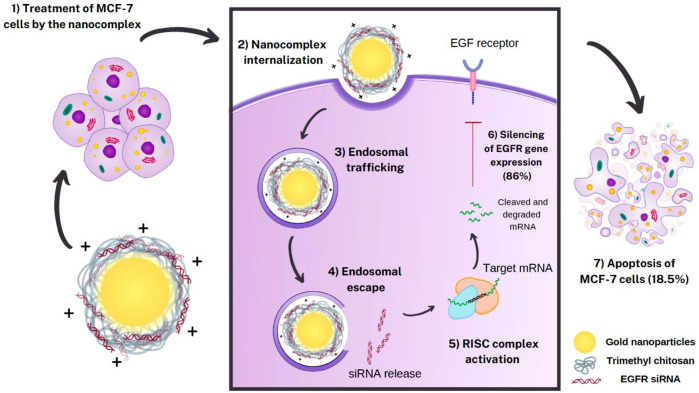

## Introduction

Breast cancer is the leading cause of cancer-related burden of disease in women (Britt et al., 2020). Based on molecular subtypes, breast cancer can be categorized as luminal A, luminal B, human epidermal growth factor receptor 2 (HER2)-enriched, and triple-negative. Triple-negative breast cancer is characterized by a lack of expression of progesterone receptor (PR), estrogen receptor (ER) and HER2, and has poor prognosis (Zubair et al., 2021). Conventional therapeutics for this type of breast cancer include chemotherapy, surgery and radiotherapy. Despite recent advances in discovery of effective chemotherapeutic agents, challenges such as toxic side effects and induction of drug resistance are yet to be fully addressed (Nedeljković and Damjanović, 2019). Therefore, targeted drug delivery to the tumor tissue in combination with therapeutic biomolecules have been explored in recent years (Zhang et al., 2021).

Small interfering RNA (siRNA), as a post transcriptional regulation of gene expression, has been introduced for fundamental treatment of mutant genes in cancer. siRNAs are double-stranded non-coding RNAs generally between 20 and 25 base pairs in length that can interact with the RNA-induced silencing complex (RISC), to degrade and cleave a complementary mRNA (Feng et al., 2021). Although many studies have shown specific gene silencing using siRNA, its delivery *in vivo* can be challenging due to various biological barriers. Nuclease degradation, endosomal trapping, rapid and extensive renal excretion, and limited cellular uptake, are some of the limitations that siRNAs face to exert their therapeutic effect (White, 2008; Mishra et al., 2017). Other major challenges for siRNA-based cancer therapeutics include controlling off-target effects of siRNA by increasing the specificity of the siRNA and avoiding unwanted immune responses (Bartoszewski and Sikorski, 2019). To overcome these challenges, it is essential to use a strategy to deliver siRNA effectively (Guo et al., 2021).

Over-expression of epidermal growth factor receptor (EGFR) mainly in triple negative breast cancer is known to promote tumor survival and suppress apoptosis (Lo et al., 2006). EGFR belongs to HER or ErbB family, which consists of four membrane-bound tyrosine kinase receptors (Lo et al., 2006; Yu et al., 2018). Using siRNA to knockdown the expression of EGFR genes can suppress the growth of breast cancer cells. In a study, silencing of EGFR1 and ERBB2 genes with siRNAs delivered by carbonate apatite nanoparticles (NPs), induced apoptotic cell death and decreased the tumor burden in mice (Tiash et al., 2017).

We employed Gold nanoparticles (AuNPs) for efficient packaging and delivery of siRNA against EGFR, as AuNPs are good candidates for nucleic acid delivery due to their distinctive features including simple synthesis enabling the control of size and shape, Surface Plasmon Resonance (SPR), easy functionalization, and biocompatibility (Boca et al., 2011; Han et al., 2012; Zhao et al., 2012). Uncoated AuNPs tend to aggregate easily because of the highly reactive free electrons on their surface. In addition to increased stability, surface modification of AuNPs with ligands also increases the nanoparticle affinity to targeted cells (Boca et al., 2011; Muddineti et al., 2015; Sanità et al., 2020).

We chose a chitosan derivative to coat the AuNPs. Chitosan has favorable properties as a drug delivery carrier. As a natural biopolymer derived from chitin it is relatively abundant. It is composed of β-1,4 linked N-acetylated D-glucosamine (GlcNAc; A-unit) and D-glucosamine (GlcN; D-unit). Chitosan is biocompatible and biodegradable and possesses low toxicity without considerable immunologic reactions (Chaharband et al., 2020). However, chitosan is only soluble in acidic media and practically insoluble at higher pH values. Thereby, we included trimethyl chitosan (TMC) as a permeation enhancer and positively charged polymer (Mourya and Inamdar, 2009; Wu et al., 2016) for surface modification of the AuNPs. TMC is a biocompatible quaternary ammonium derivative of chitosan. A persistent positive charge makes TMC a strong mucoadhesive polymer (M Ways et al., 2018). TMC is also soluble in acidic, basic, or neutral pHs. TMC has shown promising results in DNA delivery and is widely reported as a safe and efficient absorption enhancer (Mourya and Inamdar, 2009; Wu et al., 2016).

Herein, we used a safe and simple nano-based delivery system to carry EGFR-siRNA to breast cancer cells. Positively charged TMC coated AuNPs, acting as nanocarriers, interact with negatively charged siRNA via electrostatic interaction and form complexes. These complexes showed were readily uptaken by the cancer cells, knocked down the expression of the targeted gene, suppressed cell growth and induced apoptosis.

## Materials and Methods

### Materials

HAuCl4 was purchased from Sigma-Aldrich (Overijse, Belgium). Chitosan (110–150 kDa, 95% degree of deacetylation) was provided by Primex (Karmøy, Norway). Ascorbic acid was obtained from AppliChem (Darmstadt, Germany). The 3-(4,5-dimethylthiazol-2-yl)-2,5-diphenyl tetrazolium bromide (MTT reagent) was purchased from Sigma-Aldrich (Mannheim, Germany). Dulbeccoʼs modified Eagleʼs medium (DMEM) high glucose, fetal bovine serum (FBS), and penicillin-streptomycin were obtained from Biosera (Nuaille, France). EGFR siRNA was procured from Invitrogen ThermoFisher Scientific (California, United States of America). The MCF-7 breast cancer cell line was acquired from the National Cell Bank of Iran at Pasteur Institute (Tehran, Iran). The Cy-5 conjugated oligonucleotide was provided by Macrogen (Korea). Annexin V-FITC kit was purchased from Miltenyi Biotec (Germany).

### Methods

#### Synthesis of TMC-Coated AuNPs

AuNPs were synthesized using the method reported by Boca et al. 5 ml of 0.5 × 10^–3^ M tetrachloroauric acid (HAuCl4) solution was stirred at room temperature for 5 min. Then, to reduce the gold salt, 1 ml of a freshly prepared solution of ascorbic acid (7.5 × 10^–3^ M) was added. The mixture instantly and rapidly turned colorless, dark blue, and pinkish-red, respectively. The final pinkish-red color showed the formation of the AuNPs (Boca et al., 2011).

TMC was synthesized according to the previously reported protocol (Atyabi et al., 2005; Hajiramezanali et al., 2019). Briefly, chitosan was dispersed in N-methyl-2-pyrrolidone at 60°C followed by the addition of sodium iodide, sodium hydroxide aqueous solution, and methyl iodide, and the mixture was stirred for 5 h at 60°C. The product (TMC iodide) was precipitated and washed with acetone. The sediment was dissolved in sodium chloride aqueous solution to exchange the iodide ions with chloride ions. The final solution was dialyzed against distilled water for 1 day and finally lyophilized.

TMC-coated AuNPs (AuNPs-TMC) were prepared by adding 1 ml of prepared TMC solution (2 mg/ml) in deionized water to AuNPs while continuing the stirring process for another 15 min. The final solution was centrifuged at 8000 rpm, 25°C for 20 min to remove unconjugated TMC. All glassware used was cleaned with freshly prepared Aqua Regia solution (HCl:HNO3 3:1) and then rinsed thoroughly with deionized water before use.

#### Preparation of AuNPs-TMC/siRNA Nanocomplex

AuNPs-TMC/siRNA nanocomplex was prepared as follows: Briefly, AuNPs-TMC were mixed with EGFR-siRNA (100 µM) at different weight ratios (7:1, 10:1, and 21:1 w/w) and incubated for 2 h at 25°C for electrostatic complexation of negatively charged siRNA to the positively charged TMC layer of AuNPs-TMC.

#### Characterization of AuNPs

To assess the chemical structure of TMC, Fourier transform infrared (FTIR) spectroscopy (Spectrum two, PerkinElmer, United States) was carried out. The degrees of quaternization (DQ) and dimethylation (DD) of TMC were calculated using 1H-nuclear magnetic resonance (1H-NMR) spectrum (Avanace 500 MHz; Bruker, Rheinstetten, Germany), obtained in D2O as a solvent.

The AuNPs and AuNPs-TMC were analyzed by ultraviolet–visible spectrophotometry (UV–Vis spectrophotometer, CE7500; Cecil, Cambridge, United Kingdom). The UV–Vis scan was conducted at 400–800 nm wavelengths.

The average size, polydispersity index (PDI), and surface zeta potential of the nanoparticles and nanocomplex were measured by dynamic light scattering using Zetasizer (Nano-ZS; Malvern Instruments, Malvern, United Kingdom) with a wavelength of 633 nm at 25°C with 90°C scattering detector angle. Zeta potential measurements were obtained using laser Doppler electrophoresis by Zetasizer.

The morphology of nanoparticles was assessed by scanning electron microscopy (SEM; MIRA3; TESCAN, Czech Republic) and transmission electron microscopy (TEM) (Zeiss-EM10C-80 kV; Oberkochen, Germany).

#### Gel Retardation Assay of AuNPs-TMC/siRNA

The complexation efficiency of siRNA to nanoparticles was assessed by agarose gel-electrophoresis at different w/w ratios of AuNPs-TMC/siRNA. In this manner, the electrophoretic mobility of samples on 2% (w/v) agarose gel was carried out for 20 min at 100 V in 1 M Tris-acetate-EDTA running buffer with pH  =  8, and the bands were stained with GelRed.

#### *In Vitro* siRNA Release

To investigate the release profile of siRNA from the nanoparticles, AuNPs-TMC were complexed with siRNA at a w/w ratio of 10:1 and incubated in aqueous media containing 10% FBS at 37°C. Then, at different time points, the suspension was centrifuged at 5000 g for 10 min, and after removal of the 1 μL of the supernatant, the concentration of siRNA was measured by Nanodrop spectrophotometer (Thermo Scientific, United States) at 260 nm.

#### *In Vitro* Cellular Studies

##### Cellular Uptake Study

To study the cellular uptake of AuNPs-TMC, Cy5-labeled negative control siRNA was used. Cy5-labeled siRNA (2 nM) was added to AuNPs-TMC (56 μg/ml) and vortexed for 1 min at 2,000 rpm and further, it was incubated for 2 h at 25°C in the dark condition to make AuNPs-TMC/Cy5-labeled siRNA.

The cellular uptake of AuNPs-TMC/Cy5-labeled siRNA was evaluated using confocal laser-scanning microscopy (Nikon, Japan) and also a flow cytometry technique (FACSCalibur; BD Biosciences). MCF-7 breast cancer cells were seeded in a six-well plates (2 × 10^5^ cells/well) in DMEM medium containing 10% FBS and incubated for 24 h at 37°C in 5% CO_2_. Cells were treated with AuNPs-TMC/Cy5-labeled siRNA and incubated for 4 h.

For confocal microscopy, after 4 h incubation, the cells were fixed by formaldehyde solution (1 ml, 4% v/v) and the nucleus was stained by DAPI. Finally, the cellular uptake of the nanoparticles was evaluated by confocal microscopy.

For flow cytometry analysis, after 4 h incubation, cells were washed with phosphate-buffered saline (PBS; pH 7.4) and trypsinized. After centrifuging and washing the cells with PBS twice, the cells were resuspended in the PBS. The percentage of cellular uptake was evaluated by flow cytometry and analyzed by FlowJo software (Flowjo 10.0.9).

##### Cell Viability Assay

The cytotoxicity of nanoparticles was evaluated in MCF-7 breast cancer cell line using MTT assay. Cells were seeded in 96-well plates (5×10^3^ cells/well) in DMEM cell culture media containing 10% FBS. After 24 h, cells were treated with AuNPs-TMC, free EGFR-siRNA and AuNPs-TMC/EGFR-siRNA (28, 56 and 112 μg/ml of AuNPs-TMC, and 50, 100, and 200 nM siRNA). The w/w ratio of AuNPs-TMC/EGFR-siRNA was 10:1 in all samples. Later, cells were further incubated for 48 h. Then, the surface media of each well was replaced by MTT solution and incubated for 2 h. The formazan crystals were dissolved by dimethyl sulfoxide (DMSO) and the optical density was measured by an ELIZA plate reader at 570 nm (Plate reader EL×800; BIOTEC). A reference wavelength of 630 nm was used.

##### Apoptosis Assay

To assess the apoptosis level, MCF-7 cells were seeded in six-well plates (2×10^5^ cells/well) in DMEM containing 10% FBS. After 24 h incubation, cells were treated with AuNPs-TMC, free EGFR-siRNA and AuNPs-TMC/EGFR-siRNA (at the concentrations of 100 nM for EGFR-siRNA and 56 μg/ml for AuNPs-TMC) and were further incubated for 24 h. Then, the cells were collected by adding 0.25% trypsin-EDTA and were washed with PBS twice and resuspended in 150 µl of binding buffer. The untreated cells were used as controls. Thereafter, 5 µl FITC-conjugated Annexin-V and propidium iodide (PI) were added to the cells, vortexed, and incubated at 25°C in the dark for 10 min. Apoptosis level was measured by flow cytometry analysis.

##### Gene Expression Analysis

To evaluate the mRNA expression level of the EGFR gene in MCF-7 cells, a real-time polymerase chain reaction (RT-PCR) was performed. First, MCF-7 cells were seeded and treated with AuNPs-TMC/EGFR-siRNA as same as the apoptosis protocol described above. After 24 h incubation, the whole RNA obtained from MCF-7 cells were extracted using an RNA extraction kit (RiboEx, Korea). A cDNA Synthesis Kit (biofact, Korea) was used to generate cDNA. The RT-PCR reinforcement was carried out by AddScript RT-PCR SYBR Master (2x conc.) (Addbio, Korea). The primer sequences are presented in Table 1.

**TABLE 1:** The primer sequences utilized in real-time PCR.

Forward sequence	AAC​ACC​CTG​GTC​TGG​AAG​TAC​G
Reverse Sequence	TCG​TTG​GAC​AGC​CTT​CAA​GAC​C

PCR, polymerase chain reaction.

## Results and Discussion

### Characterization of TMC

**FIGURE 1:**
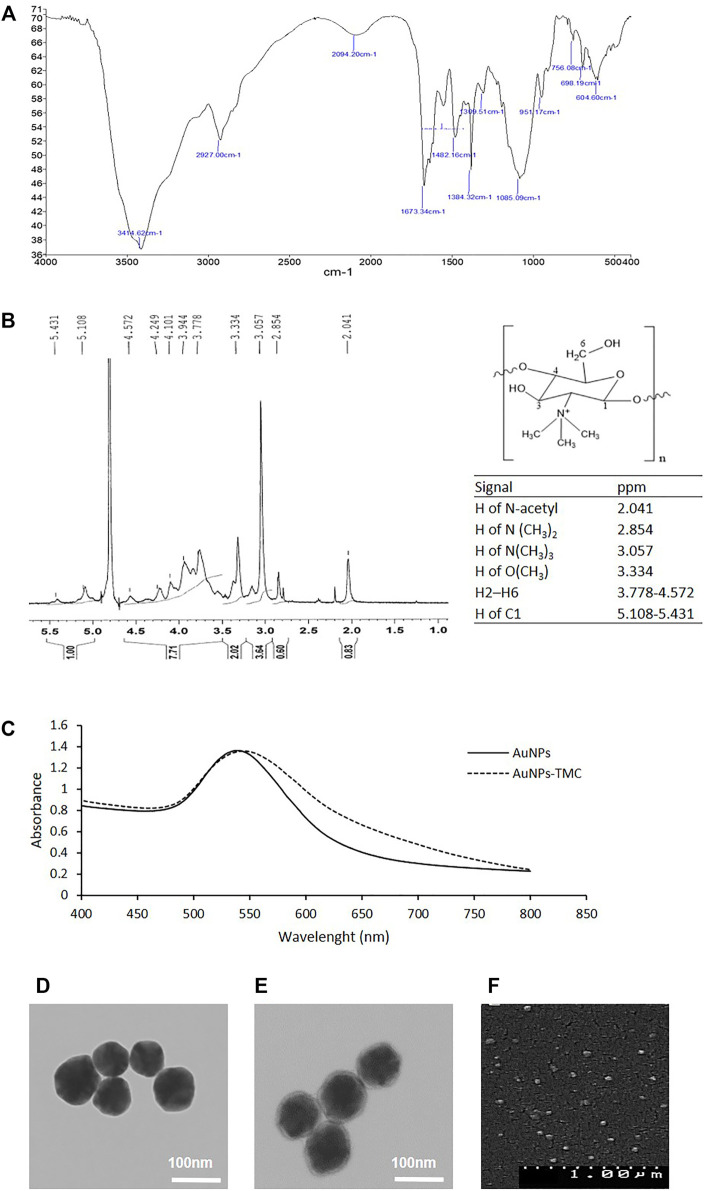
Synthesis and characterization of the AuNPs-TMC: **(A)** FTIR spectrum of TMC. **(B)** NMR Spectrum of TMC. **(C)** UV–Vis spectrum of AuNPs and AuNPs-TMC. **(D)** TEM image of AuNPs. **(E)** TEM image of AuNPs-TMC. **(F)** SEM image of AuNPs-TMC.

Chemical structure of TMC was elucidated using FTIR and 1H-NMR analysis. Figure 1A illustrates the FTIR spectrum of TMC. We observed the characteristic peaks at 3414 cm^−1^ (O-H/N-H stretching), 2927 cm^−1^ (C–H stretching, pyranose ring), 1673 cm^−1^ (C=O stretching of amide I, NH-Acetyl group), 1554 cm^−1^ (N-H bending, amine group), and 1085 cm^−1^ (C-O/C-N stretching). The peaks at 2927 cm^−1^ and 1482 cm^−1^ indicate the CH2 group and the peaks at 2880, and 1384 show the CH3 group (Geçer et al., 2010; Martins et al., 2011; Chatterjee et al., 2019).

Figure 1B depicts the 1H-NMR spectrum of TMC, the chemical structure of TMC, and the related signals. Signals at 2.854 and 3.057 ppm correlated to the protons of N(CH3)2 (DD = 12.8%) and N(CH3)3 (DQ = 51.2%) groups, respectively. The signal at 3.334 ppm can be attributed to the OCH3 protons in TMC polymers; because during the synthesis of TMC, methylation of the hydroxyl groups and formation of OCH3 groups is also possible (Mourya and Inamdar, 2009; Hu et al., 2013; Pardeshi and Belgamwar, 2016).

### Characterization of AuNPs

AuNPs were synthesized by a low-cost and rapid method of direct reduction of aqueous HAuCl4 solution using ascorbic acid at room temperature (Sau et al., 2001). The high water solubility, low toxicity, and biodegradability of ascorbic acid made it a naturally accessible reducing agent. Herein, ascorbic acid acts both as a reducing agent and stabilizer by charging particles through ion adsorption (Boca et al., 2011). The formation of AuNPs was revealed by turning the slightly yellow solution into a colloid pinkish-red color during synthesis, which is related to the Surface Plasmon Resonance (SPR) of AuNPs and was confirmed by UV-Vis spectroscopy (Sztandera et al., 2018). Figure 1C shows the extinction spectrum of AuNPs with an SPRmax = 540 nm which is in agreement with the maximum adsorption of spherical gold nanoparticles (Huang et al., 2006).

AuNPs-TMC were prepared by the addition of TMC to AuNPs. The positive charge of quaternary ammonium in the TMC structure enabled the electrostatic interaction with the negative surface potential of AuNPs (dos Santos et al., 2004). As shown in Table 2, AuNPs had a hydrodynamic diameter of about 60 nm with a negative surface charge; while, coating of TMC on the AuNPs core resulted in slight increase in particle size (67 nm) and positive surface charge (+45 mV). The formation of TMC coated AuNPs was also assessed by UV-Vis spectroscopy. As shown in Figure 1C, the spectrum of AuNPs-TMC with SPRmax of 544.5 nm showed a redshift and broader spectrum in comparison to AuNPs which was attributed to both the effects of the polymer coating and the increased particles size. No aggregation occurred following the addition of the TMC, evidenced by the SPR spectrum of AuNPs-TMC, and we observed no significant change in the color and clarity of the colloidal solution during synthesis. This synthesis method produced nanoparticle with long-term stability without any sign of agglomeration (Boca et al., 2011).

**TABLE 2:** Size, PDI, and zeta potential of NPs.

Nanoparticles	Size (nm)	PDI	Zeta Potential (mV)
AuNPs	60 ± 5	0.228 ± 0.012	−4.1 ± 2.3
AuNPs-TMC	67 ± 7	0.27 ± 0.023	+45.4 ± 4.7
AuNPs-TMC/siRNA (10:1 w/w ratio)	82 ± 5	0.265 ± 0.019	+11.4 ± 1.8

NP: nanoparticle, PDI: polydispersity index, TMC**:** trimethyl chitosan.

Data are shown as mean ± SD (*n* = 3).

The TEM and SEM images of AuNPs-TMC (Figures 1E,F) show spherical morphology of AuNPs-TMC with an average particle size of ∼70 nm and uniform dispersity without aggregation, which is in agreement with data measured by DLS. A soft halo around the Au core of AuNPs-TMC with ∼4 nm thickness compared to TEM image of AuNPs (Figure 1D) also revealed the successful coating of TMC. The TMC outer layer can prevent the interactions between nanoparticles through steric and electrostatic stabilization and improves stability (Kulkarni et al., 2017).

According to Table 2, AuNPs-TMC/siRNA (10:1 w/w ratio) showed an increased particle size and also a reduction in zeta potential (from +45 to +11 mV) in comparison to blank AuNPs-TMC. These data also confirmed the successful complexation of siRNA with AuNPs-TMC.

### siRNA Efficiently Interacts With AuNPs-TMC

**FIGURE 2:**
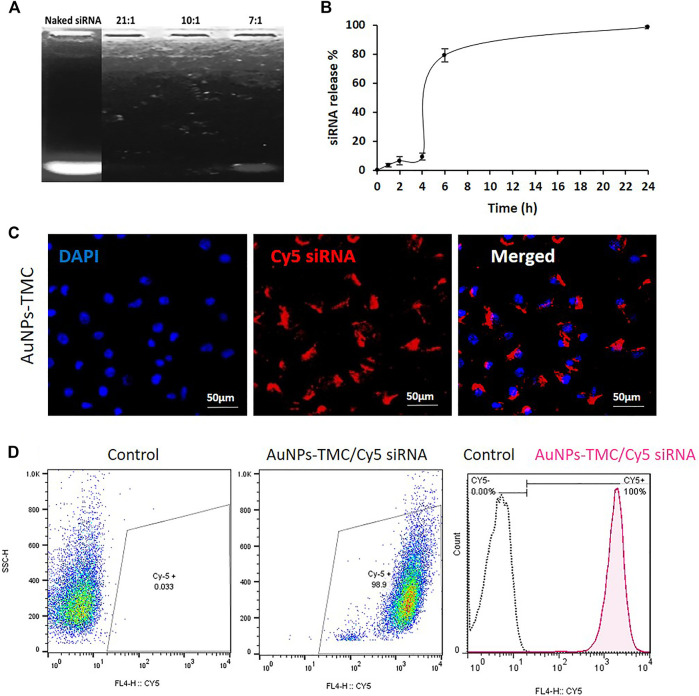
Stability, release profile and cellular uptake of AuNPs-TMC/siRNA. **(A)** Agarose gel electrophoresis for naked siRNA and different w/w ratios of AuNPs-TMC/siRNA. **(B)** Release Profile of siRNA from AuNPs-TMC/siRNA in 10:1 w/w ratio in 10% FBS aqueous media at 37°C. The percentage of free siRNA was measured at 0, 1, 2, 4, 6, and 24 h time points, data are presented as mean ± SD of triplicates. Cellular uptake of AuNPs-TMC/Cy5-labeled siRNA (w/w ratio 10:1) in MCF-7 cells after 4 h incubation. **(C)** Confocal microscopy images (The cell nuclei were stained by DAPI). **(D)** Flow cytometry analysis; which is shown in histogram with the X-axis indicating the mean fluorescence intensity and the Y-axis indicating the cell count.

Agarose gel electrophoresis was conducted to assess the loading capacity of AuNPs-TMC to condense and package siRNA. As depicted in Figure 2A, compared to naked siRNA, at 21:1 and 10:1 w/w ratio of AuNPs-TMC/siRNA, no siRNA-related band was observed and siRNA migration was completely retarded which implied that AuNPs-TMC could retain siRNA at these w/w ratios. However, the AuNPs-TMC were not able to retard the migration of siRNA at the 7:1 w/w ratio.

TMC, as a cationic polysaccharide, can form a polyelectrolyte complex with nucleotide strands and proteins (Kulkarni et al., 2017). EGFR-siRNA molecules with negative phosphate groups could bind very efficiently to the positively charged surface of AuNPs-TMC *via* electrostatic interactions in an optimized weight ratio. Therefore, 10:1 w/w ratio of AuNPs-TMC/siRNA was used as the optimum binding ratio for further assays.

### *In Vitro* Release of siRNA

The *in vitro* release profile of siRNA from the NPs is shown in Figure 2B. The siRNA release showed a three-step sigmoidal release profile. First, approximately 9% of siRNA was released over 4 h, which is related to the sustained release of the surface-loaded siRNA (Cun et al., 2011). Second, a sudden release of siRNA at 6 h was seen, until the cumulative release of siRNA reached 79%. The remaining siRNA was completely released within 24 h. As a window of 6 h is an appropriate time for cargo to reach the tumor tissue in an intravenously injected nanocarrier, this sudden release after 6 h could be a favorable aspect. TMC has good water solubility in neutral pH environments (Kulkarni et al., 2017). Therefore, the sudden siRNA release at 6 h could be related to aqueous solubilization of the surface layers of the TMC shell which facilitated the sudden release of siRNA. The use of 10% FBS in the release media increased the siRNA release. Negatively charged proteins, present in FBS, have been reported to competitively bind to nanocarriers and enhance release (Ju and Yeo, 2012; Wang et al., 2014). Xu et al. (2021), also reported that siRNA release from nanodiamonds was increased by enhancing the FBS percentage from 1.25% to 10% in the release media. In the intracellular environment, the presence of various soluble biological polyanions can competitively replace and dissociate the nucleic acids from nanocomplexes (Cozzolino et al., 2021).

siRNA dissociation from the nanocomplex in the cytoplasm is an important step for successful siRNA-mediated knockdown (Dominska and Dykxhoorn, 2010). This release profile for AuNPs-TMC/siRNA could be a potential property for the designed nanocomplexes. Because, if the nanocomplexes reach their target breast cancer cells before 4 h, the sudden release of the gene inside the cellular endosome or cytoplasm will increase the efficiency of the EGFR-siRNA transfection and also prevent its plasma degradation.

### Efficient Cellular Uptake of AuNPs-TMC/siRNA Into Breast Cancer Cells

Effective silencing of the target EGFR mRNA depends on successful internalization of NPs by cancer cells with subsequent release of siRNA in the cytoplasm (Tiash et al., 2017). Confocal microscopy and flow cytometry analysis were performed for qualitative and quantitative evaluation of the cellular uptake of AuNPs-TMC/CY5-labeled siRNA into MCF-7 breast cancer cells. According to confocal images (Figure 2C), after 4 h incubation, the nanocomplex was effectively internalized into the MCF-7 cells and localized inside the cytoplasm, where siRNA mediates its function by interaction with the RNA interference machinery (Song et al., 2010). The result of flow cytometry analysis (Figure 2D) also confirmed the successful and complete cellular uptake of the nanocomplex after 4 h incubation. It has been previously shown that maximum uptake occured for AuNPs in the range of about 50 nm spheres (Sztandera et al., 2018). Our designed Au-nanocomplex with an approximate 70 nm size also showed high cellular uptake. The AuNPs exhibited excellent siRNA packaging capability. We also observed increased siRNA loading capacity with lower NP to siRNA w/w ratio compared to previous studies (Darvishi et al., 2013).

Cellular uptake of NPs is strongly associated with their surface characteristic, mainly zeta potential (Foroozandeh and Aziz, 2018). In principle, the cationic nanoparticles are more effective in binding with anionic sulfated proteoglycans molecules in the lipid bilayer of mammalian cells (Panariti et al., 2012). Thereby, the positive charge of TMC, in addition to increasing the oligonucleotide loading capacity, also increased the cellular uptake. However, it should be noted that very high positive surface charge of NPs may be toxic for cells (Zhao et al., 2012). Our designed AuNPs-TMC/siRNA by having a moderate positive zeta potential of about +11 mV showed high cellular uptake. We expect that the TMC coated AuNPs undergo adsorption-mediated endocytosis and clathrin-dependent endocytosis (Sheng et al., 2015). AuNPs-TMC are thus capable of carrying the electrostatically linked siRNA into the breast cancer cells, after 4 h of incubation, through endocytosis as a non-viral vector and finally, by the sudden release of the bound siRNA into the cytosol would lead to appropriate gene silencing (Sun et al., 2021).

### Efficient Inhibition of Breast Cancer Cell Proliferation by AuNPs-TMC/EGFR-siRNA

**FIGURE 3:**
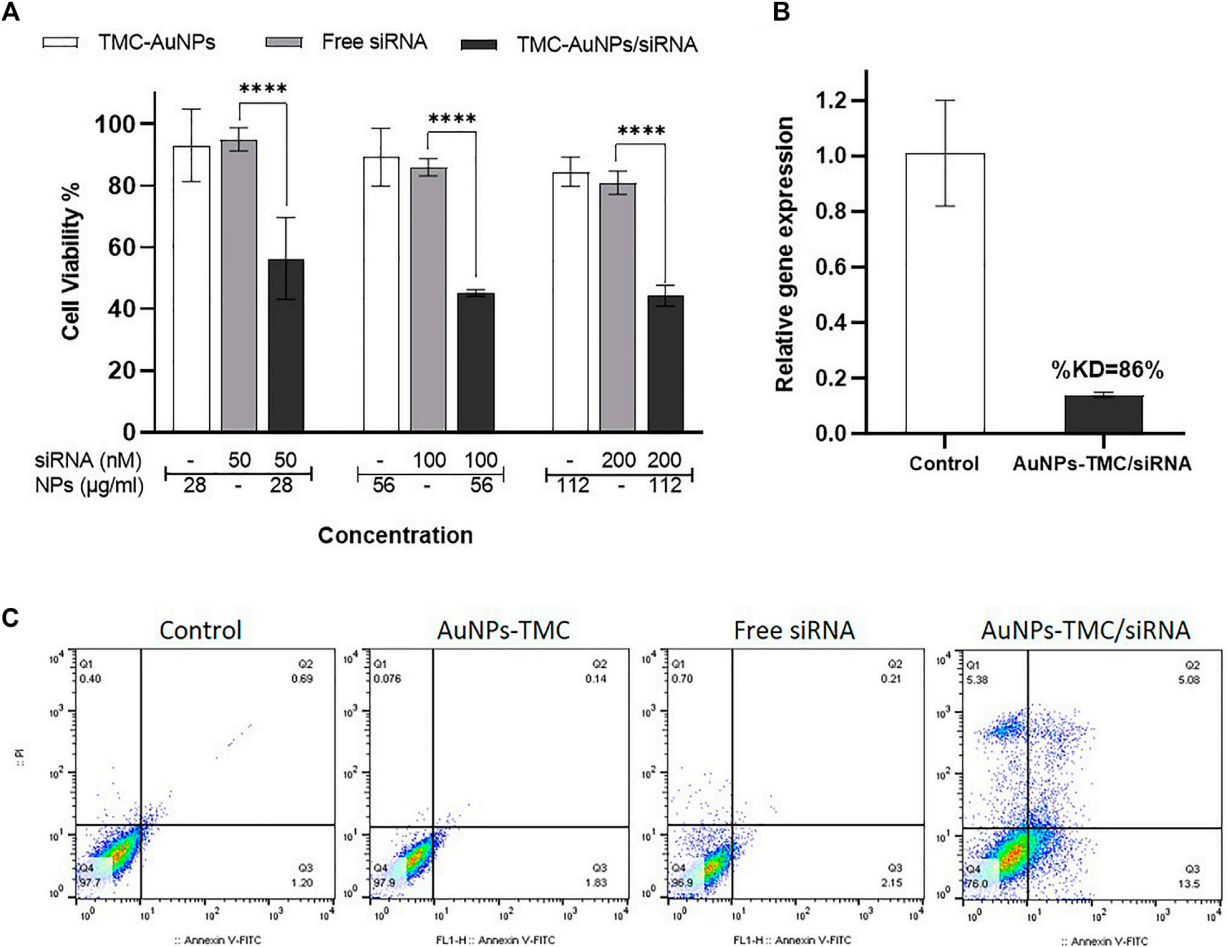
Therapeutic effects of AuNPs-TMC/siRNA. **(A)** Cell viability percentage of MCF-7 cells after 48 h incubation with AuNPs-TMC, Free siRNA and AuNPs-TMC/siRNA (w/w ratio 10:1) at different concentration of NPs (28, 56 and 112 μg/ml) and siRNA (50, 100 and 200 nM). Data is shown as mean ± SD (N = 3). Statistical analysis: Two-way ANOVA, post-test Sidak, (***p < 0.001 significantly difference between columns). **(B)** Relative gene expression percent of EGFR in MCF-7 cells after 24 h incubation with AuNPs-TMC/EGFR-siRNA in w/w ratio of 10:1 (56 μg/ml/100 nM) using RT-PCR method. (KD = knockdown). **(C)** Apoptosis assay in MCF-7 cancer cells following no treatment and treatment with AuNPs-TMC, Free siRNA and AuNPs-TMC/EGFR-siRNA (56 μg/ml/100 nM) after 24 h using annexin V-FITC/PI staining.

The MTT assay was carried out to evaluate the effect of AuNPs-TMC, free siRNA and AuNPs-TMC/siRNA on inhibition of MCF-7 cells proliferation after 48 h incubation. As demonstrated in Figure 3A, AuNPs-TMC proved to be safe and nontoxic, and no significant decrease in cell viability occurred as the concentration increased from 30 to 120 μg/ml (84.5% cell viability in the highest concentration). However, in AuNPs-TMC/siRNA nanocomplexes (w/w ratio 10:1) with different concentrations of EGFR-siRNA (50, 100, and 200 nM), a significant cell growth inhibition was demonstrated to about 50% (*p* < 0.001) in comparison to free siRNA with the same siRNA concentration. As AuNPs-TMC were safe and non-toxic, this high and efficient cell growth inhibition was related to the successful transfection of EGFR-siRNA and confirmed the therapeutic potential of AuNPs-TMC/EGFR-siRNA for breast cancer treatment. By increasing the siRNA concentration from 50 to 100 nM, the cell viability decreased from 56 % to 45%, and an additional increase in siRNA concentration had no greater effect on cell growth inhibition. Therefore, the 100 nM siRNA concentration was selected for further cellular analysis.

### AuNPs-TMC/EGFR-siRNA Enhanced Apoptosis in Breast Cancer Cells

The efficacy of AuNPs-TMC/EGFR-siRNA (w/w 10:1) was also validated by cell apoptosis assay of MCF-7 cells after 24 h incubation, using flow cytometry based on annexin V-FITC/PI staining. As displayed in Figure 3C, control cells that were not treated presented a cell apoptosis rate of 1.89%. AuNPs-TMC and free siRNA showed 1.97% and 2.36% apoptosis rate, while cells treated with AuNPs-TMC/EGFR-siRNA (56 μg/ml/100 nM) showed high apoptosis rate of 18.58% (early and late stages in total). This result is in agreement with the high cellular uptake and high cell growth inhibition of AuNPs-TMC/EGFR-siRNA. Overexpression of EGFR promotes tumor survival and also suppresses apoptosis of breast cancer cells (Arteaga and Engelman, 2014). Thereby, as a therapeutic approach, efficient inhibition of EGFR expression could result in enhanced tumor suppression by the caspase-7-mediated apoptotic pathway (Tiash et al., 2017). Thus, high levels of apoptosis rate in the treated MCF-7 cells confirmed the potential therapeutic effect of AuNPs-TMC as a novel and simple vector for efficient delivery of EGFR-siRNA into tumor cells.

### Efficient Gene Silencing by AuNPs-TMC/EGFR-siRNA Nanocomplexes

EGFR is a transmembrane tyrosine kinase protein that is overexpressed in breast cancer and plays an important role in cell proliferation, metastasis, angiogenesis, apoptosis inhibition, and tumor progression (Jones et al., 2006; Masuda et al., 2012; Hashmi et al., 2019). Therefore, knockdown of EGFR gene expression by siRNA could induce cell death, apoptosis, and tumor cell growth inhibition. Herein, the relative percentage of EGFR gene expression was assessed by RT-PCR. As shown in Figure 3B, the relative EGFR gene expression in AuNPs-TMC/EGFR-siRNA (w/w 10:1) treated MCF-7 cells after 24 h incubation was found to be 14%, that is to say, the knockdown percentage (%KD) of gene expression was 86%. The use of siRNA nanoparticles as a therapeutic strategy has several advantages in cancer treatment. However, several factors influence the NPs/siRNA stability and half-life including nanoparticle shape, zeta potential, flexibility, surface coating, and nucleic acid loading capacity (Parvani and Jackson, 2017). The high %KD obtained by our designed AuNPs can be considered as a great success for a non-viral nucleic acid delivery system and confirmed that the designed AuNPs-TMC/EGFR-siRNA nanocomplex had good stability, a high rate of transfection and that it was able to release the siRNA in cytosol.

In many studies, complex polymers and structures have been used to achieve high transfection and gene silencing (Darvishi et al., 2013; Diaz-Dussan et al., 2017; Shaabani et al., 2021). However, to improve clinical application of these systems, the need for simpler designs that can be easily scaled up is of high interest (Zu and Gao, 2021). We proposed a simple and easily scalable NP for *in vivo* delivery of oligonucleotides that is also cost-effective.

## Conclusion

Delivery of a biological macromolecules such as oligonucleotides to the desired tissue faces many biological challenges, especially in terms of stability and targeting. To address these issues, herein AuNPs-TMC were synthesized using a simple synthesis method as a therapeutic approach for breast cancer. The NPs had spherical shape, uniform and low particle size, and were able to carry and retain EGFR-siRNA efficiently into MCF-7 cells. High cellular uptake of the nanocomplex and localization in cytosol confirmed that the system exhibited high transfection efficiency. Additionally, high cellular growth inhibition, high apoptosis rate, and significant EGFR gene knockdown in MCF-7 cells demonstrated that AuNPs-TMC were suitable carriers for siRNA delivery. Altogether, the simply prepared AuNPs-TMC can be a promising non-viral carrier for delivery of EGFR-siRNA into breast cancer cells as a therapeutic strategy for tumor growth prevention. Furthermore, the *in vivo* efficacy, safety, and biodistribution of AuNPs-TMC/siRNA nanocomplexes together with bio-imaging and phototherapy properties should be taken into account in future work to achieve a novel formulation with sufficient efficacy and low adverse effects.

## Data Availability

The original contributions presented in the study are included in the article/Supplementary Material, further inquiries can be directed to the corresponding author.
